# Supplementary material for: Randomized controlled trial for time-restricted eating in healthy volunteers without obesity
Source: Nat Commun. 2022 Feb 22;13:1003. doi: 10.1038/s41467-022-28662-5 (PMC8864028; doi:10.1038/s41467-022-28662-5)
Supplement: Supplementary file 1 — Supplementary Information [file 41467_2022_28662_MOESM1_ESM.pdf]

Table S1. Comparison of changes in metabolic health parameters among groups							
	eTRF	mTRF	control	p values			
				Among groups	eTRF vs. control	mTRF vs. control	eTRF vs. mTRF
SBP (mmHg)	-4.4 ± 9.9	-5.4 ± 9.6	0.7 ± 7.8	0.078	0.74	0.13	0.11
DBP (mmHg)	-3.4 ± 7.2	-4.7 ± 6.0	0.0 ± 8.0	0.099	0.55	0.23	0.11
MAP (mmHg)	-3.7 ± 6.9	-4.2 ± 7.8	0.2 ± 6.2	0.087	0.84	0.14	0.14
HDL (mmol/L)	-0.03 ± 0.20	-0.01 ± 0.19	0.05 ± 0.17	0.28	0.73	0.33	0.44
LDL (mmol/L)	0.00 ± 0.49	0.01 ± 0.46	-0.08 ± 0.36	0.68	0.95	0.82	0.82
TC (mmol/L)	-0.01 ± 0.57	0.04 ± 0.60	0.01 ± 0.43	0.94	0.98	0.98	0.98
TG (mmol/L)	0.02 ± 0.48	0.11 ± 0.32	0.07 ± 0.38	0.71	0.80	0.84	0.84
hsCRP (mg/L)	0.27 ± 0.68	0.07 ± 1.17	0.16 ± 0.35	0.70	0.79	0.92	0.92
ALT (U/L)	-3.2 ± 22.0	-0.2 ± 9.7	2.3 ± 14.4	0.45	0.74	0.51	0.74
ALP (U/L)	-4.0 ± 7.1	-3.4 ± 10.2	-4.9 ± 6.6	0.80	0.90	0.90	0.88
GGT (U/L)	-3.6 ± 9.0	-1.0 ± 4.5	1.0 ± 10.0	0.13	0.44	0.13	0.44
WBC (× 10^9/L)	-0.13 ± 1.29	-0.16 ± 1.56	-0.22 ± 0.89	0.96	0.99	0.99	0.99
PSQI	-1.1 ± 1.8	-0.2 ± 2.2	-0.4 ± 1.7	0.24	0.32	0.34	0.80
CNAQ	-0.2 ± 2.3	-0.8 ± 2.5	0.1 ± 1.8	0.35	0.59	0.61	0.40

Among groups p values were calculated with one-way ANOVA analysis. P-values of comparisons between two groups were calculated with Holm-Sidak's multiple comparisons test following one-way ANOVA analysis. Abbreviations: eTRF, early time-restricted feeding group; mTRF, mid-day time-restricted feeding group; SBP, systolic blood pressure; DBP, diastolic blood pressure; MAP, mean arterial pressure; HDL, high-density lipoprotein; LDL, low-density lipoprotein; TC, total cholesterol; TG, triglyceride; hsCRP, high sensitivity C-reactive protein; ALT, alanine aminotransferase; ALP, alkaline phosphatase; GGT, glutamyl-transpeptidase; WBC, white blood cells; PSQI, Pittsburgh Sleep Quality Index; CNAQ, Council of Nutrition Assessment Questionnaire.

Table S2. R-squared values of the Cosinor model plots for each participant at each testing time point

Clock genes	Group	Testing time point	R-squared values of the plot for each participant						
BMAL1	eTRF	BL	0.5311	0.02386	0.2338	0.9299	0.8669		
		FU	0.7272	0.3133	0.8833	0.4955	0.5993		
	mTRF	BL	0.4786	1	0.9773	0.9101	0.9147	0.9753	0.9718	0.5091
		FU	0.8544	0.659	0.4321	0.9978	0.01798	0.2298	0.07282	0.7702
	control	BL	0.9367	0.3223	0.925	0.9999	0.995	0.8518		
		FU	0.2455	0.5504	0.5657	0.8598	0.6983	0.9991		
SIRT1	eTRF	BL	0.3451	0.3187	0.4482	0.8802	0.8169			
		FU	0.6522	0.6195	0.5986	0.5618	0.8025			
	mTRF	BL	0.3617	0.8929	0.9908	0.9216	0.9116	0.7895	0.958	0.9007
		FU	0.4764	0.9675	0.5129	0.3101	0.615	0.4823	0.6893	0.6397
	control	BL	0.5408	0.01081	0.5701	1	0.9816	0.9999		
		FU	0.9978	0.931	0.9218	0.883	0.2242	0.8507		
CRY1	eTRF	BL	0.8275	0.3325	0.9842	0.9381	0.7458			
		FU	0.9874	0.2028	0.9911	0.989	0.7503			
	mTRF	BL	0.6052	0.919	0.9977	0.9986	0.7138	0.9985	0.8225	0.9711
		FU	0.8614	0.2869	0.59	0.9663	0.8629	0.262	0.02966	0.6667
	control	BL	0.7751	0.6526	0.9984	0.8815	0.9439	0.862		
		FU	0.7512	0.9731	0.1993	0.9372	0.955	0.7448		
CRY2	eTRF	BL	0.1537	0.8325	0.9317	0.6487	0.9928			
		FU	0.7519	0.719	0.9682	0.9454	0.6567			
	mTRF	BL	0.928	0.6472	0.7572	0.86	0.4829	0.9747	0.8224	0.8799
		FU	0.3881	0.9373	0.2372	0.5578	0.05622	0.1182	0.07135	0.2619
	control	BL	0.9534	0.6832	0.9993	0.7369	0.9534	0.7525		
		FU	0.9024	0.9806	0.4134	0.8734	0.8026	0.2539		
PER1	eTRF	BL	0.4378	0.8358	0.4995	0.3555	0.9349			
		FU	0.1167	0.6175	0.7509	0.7091	0.9454			
	mTRF	BL	0.9909	0.8837	0.9931	0.9975	0.9704	0.9822	0.8835	0.9137
		FU	0.1763	0.743	0.2292	0.7806	0.01531	0.957	0.7787	0.4172
	control	BL	0.6445	0.3386	0.452	0.8387	0.9232	0.7633		
		FU	0.9799	0.9357	0.7705	0.8259	0.2722	0.8728		
PER2	eTRF	BL	0.5022	0.1503	0.4515	0.6644	0.965			
		FU	0.277	0.7638	0.4726	0.4907	0.9627			
	mTRF	BL	0.7489	0.9862	0.9383	0.9476	0.7029	0.6252	0.9355	0.8675
		FU	0.2162	0.9521	0.8118	0.1303	0.5931	0.6696	0.9615	0.8779
	control	BL	0.7686	0.7579	0.295	0.7566	0.8687	0.6149		
		FU	0.1982	0.58	0.8098	0.9755	0.9945	0.9215		
PER3	eTRF	BL	0.3391	0.04676	0.8149	0.8073	0.2756			
		FU	0.8123	0.3054	0.7652	0.8897	0.8705			
	mTRF	BL	0.441	0.9624	0.7988	0.8412	0.9109	0.9322	0.8847	0.9999

		FU	0.4517	0.986	0.02189	0.6586	0.07802	0.3852	1	0.6586
	control	BL	0.2927	0.4874	0.9235	0.6632	0.865	0.7138		
		FU	0.7879	0.9885	0.6855	0.6649	0.4517	0.8549		

Abbreviations: BL: baseline, FU: follow-up.

Table S3. Primerss for mRNA of clock genes in PBMCs.

**Target Name**	**Primer**	
actin	F	GACAGGATGCAGAAGGAGATTACT
	R	TGATCCACATCTGCTGGAAGGT
SIRT1	F	CTACTTCGCAACTATACCCAG
	R	ATATCTCCTCGTACAGCTTC
BMAL1	F	AAAGCTTCTGCACAATCCAC
	R	CATGAGAATGCAGTCGTCCAA
CRY1	F	ACGAAAATTAAACTCCCGTCTG
	R	CTTAATAGCTGCGTCTCGTTC
CRY2	F	TGCATCTGTTGACACTCATGATTC
	R	GGTACTCCCCCAGCCCAG
PER1	F	CCTTCCCTGCCAATCCC
	R	CCTCTGCCTGTCGTCGT
PER2	F	AGCAGGTGAAAGCCAATGAAG
	R	AGGTAACGCTCTCCATCTCCTC
PER3	F	GTCCAAGCCTTACAAGCTGGTTT
	R	GACCGTCCATTTGTTGGCAT

Total RNA was pooled from PBMCs and used for cDNA synthesis. Transcript levels were then quantified by qPCR using the SYBR qPCR mix (ABI-invitrogen). Results for the respective gene of interest (SIRT1, BMAL1, CRY1, CRY2, PER1, PER2, PER3) were normalized to actin by using the 2-△△CT method. The primer sequences are presented in the table.

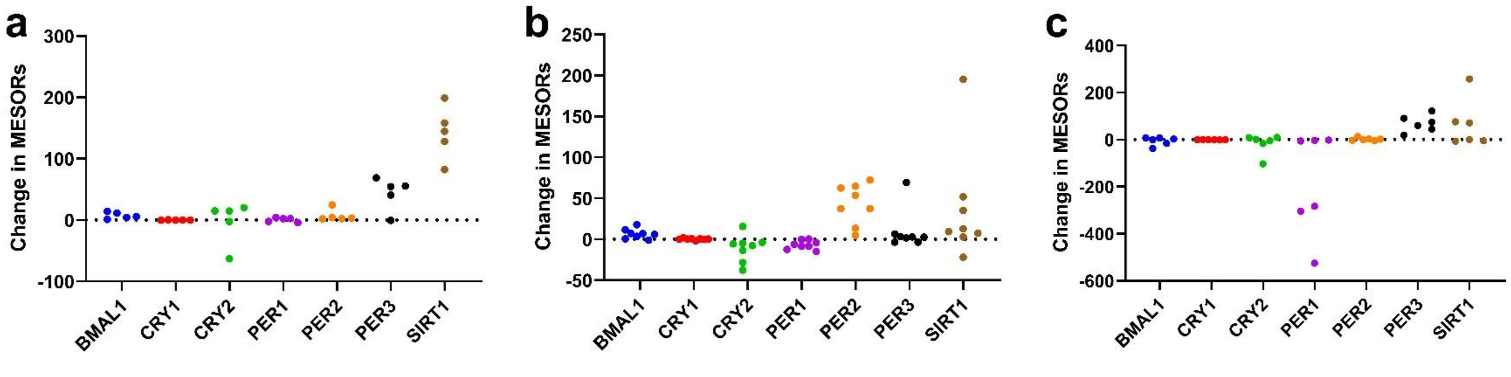

Figure S1. TRF influences the circadian rhythm MESORs of clock gene expression in peripheral blood mononuclear cells.

After Cosinor analysis, the MESORs of clock gene expression in each individual were calculated. (a) The change in MESORs of clock genes after analyzed with Cosinor analysis in eTRF group. All participants in eTRF group showed an increase in the MESORs of BMAL1, PER2, and SIRT1 after the trial. (b) The change in MESORs of clock genes after analyzed with Cosinor analysis in mTRF group. All participants in mTRF group showed an increase in the MESORs of PER2. (c) The change in amplitude of clock genes after analyzed with Cosinor analysis in control group.

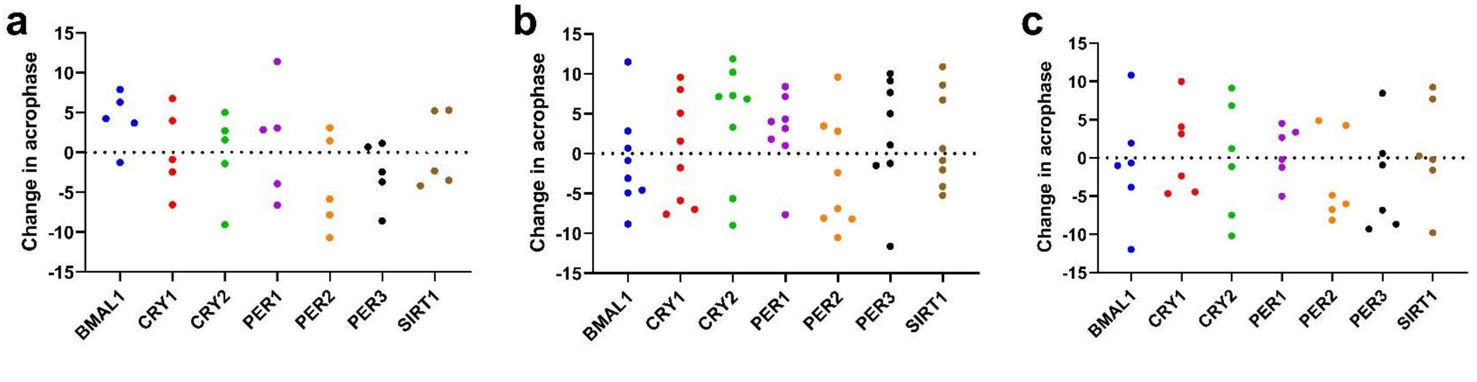

Figure S2. TRF did not influence the circadian rhythm acrophase of clock gene expression in peripheral blood mononuclear cells.

After Cosinor analysis, the acrophase of clock gene expression in each individual were calculated. (a) The change in acrophase of clock genes after analyzed with Cosinor analysis in eTRF group. (b) The change in acrophase of clock genes after analyzed with Cosinor analysis in mTRF group. (c) The change in acrophase of clock genes after analyzed with Cosinor analysis in control group.

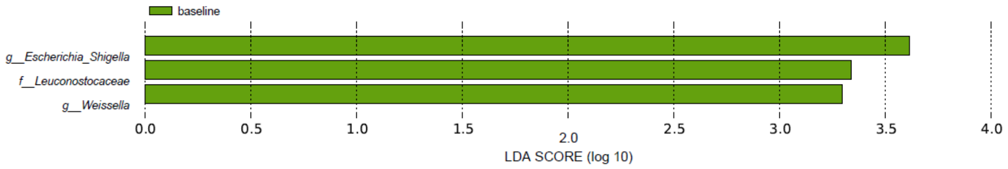

Figure S3. Linear discriminant analysis (LDA) plots of distinct taxa identified between baseline and follow-up testing results in mTRF group.

The relative abundances of *Escherichia_Shigella* and *Weissella* were enriched in baseline at genus level, and the relative abundance of *Leuconostocaceae* was enriched in follow-up at family level.

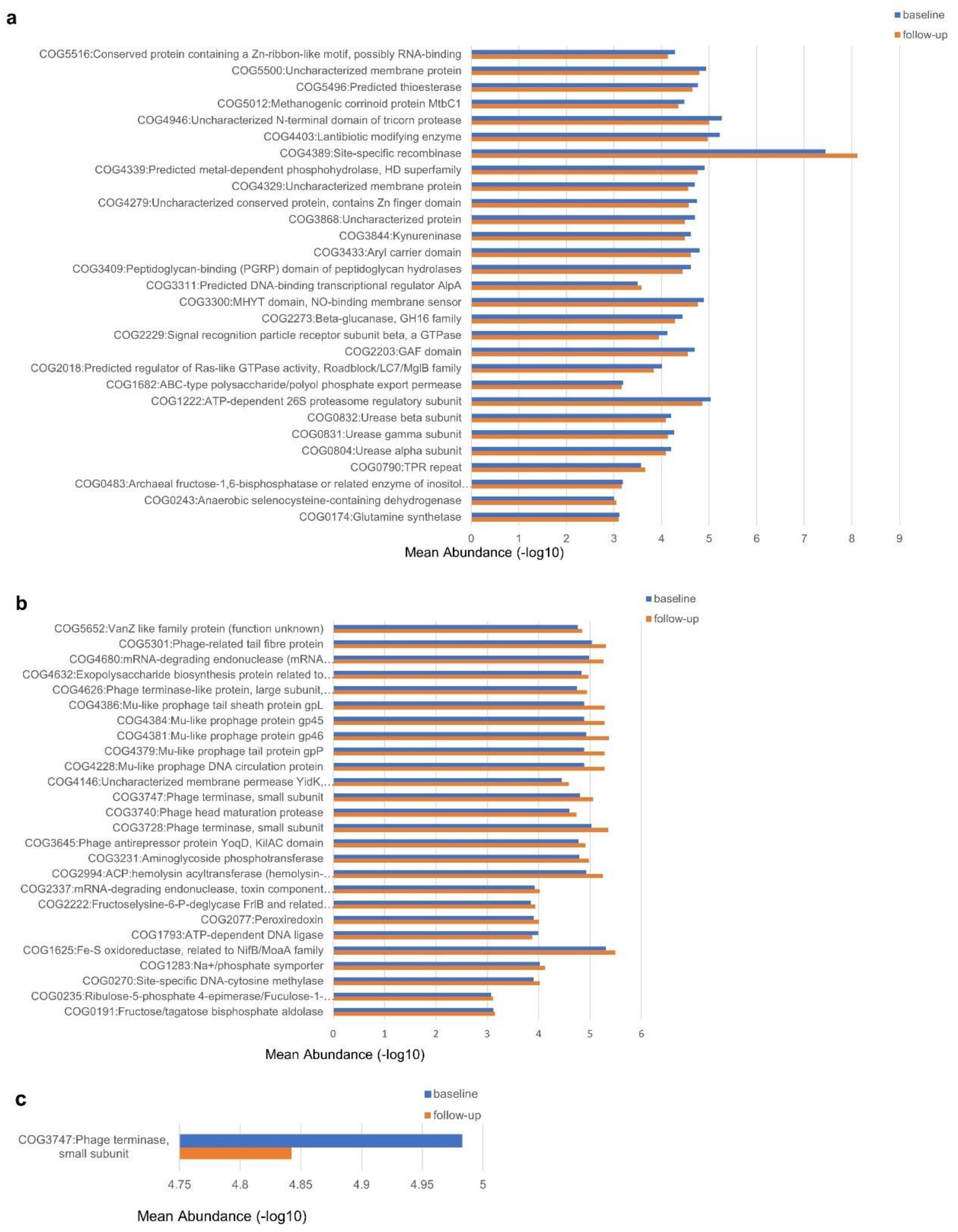

Figure S4. PICRUSt was used to analyze functional genes of microbial communities in each group. Using function predictions based on clusters of orthologous group (COG) analysis, we found 29, 26, 1 significantly different functional COGs between baseline and follow-up testing results in eTRF group (a), mTRF group (b) and control group (c), respectively.

a

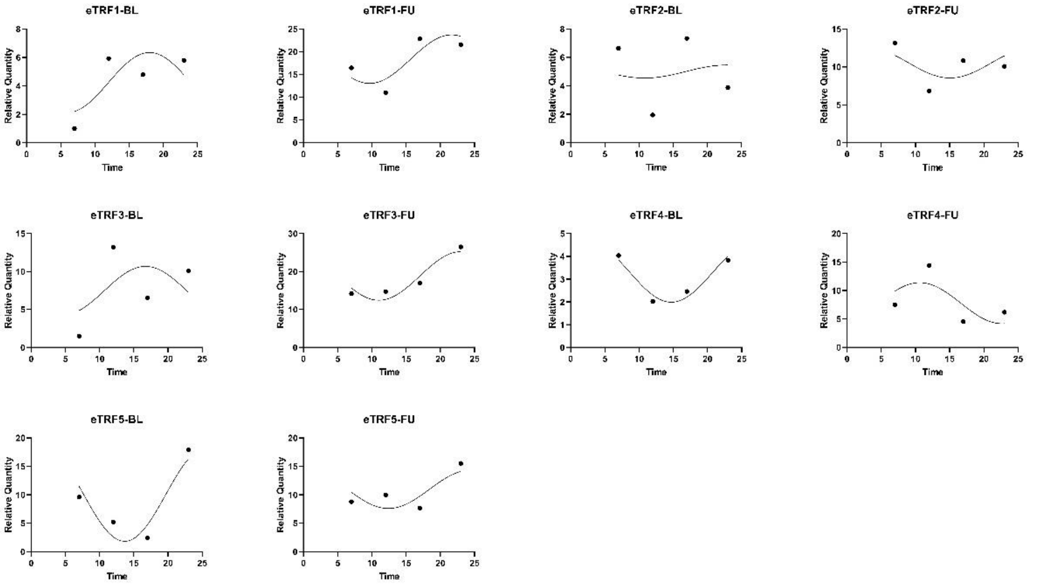
eTRF1-BL
eTRF1-FU
eTRF2-BL
eTRF2-FU
eTRF3-BL
eTRF3-FU
eTRF4-BL
eTRF4-FU
eTRF5-BL
eTRF5-FU
Relative Quantity
Time

b

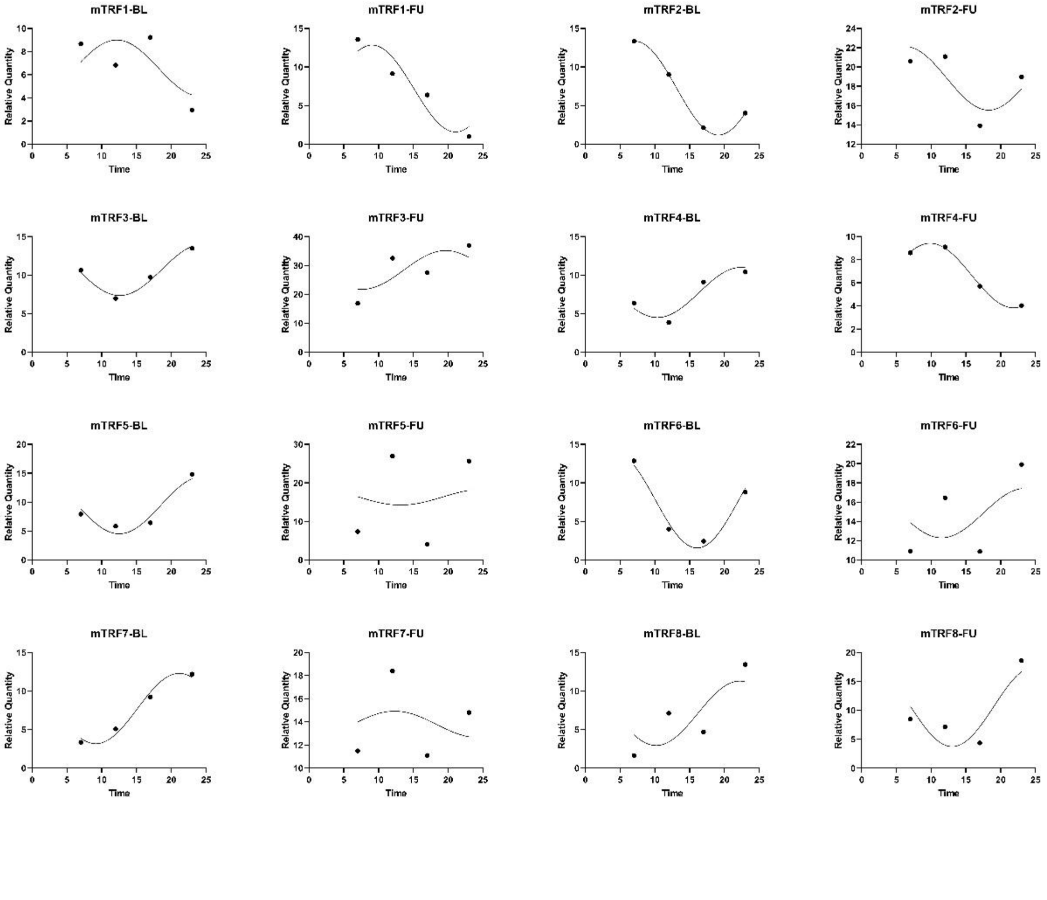
mTRF1-BL
mTRF1-FU
mTRF2-BL
mTRF2-FU
mTRF3-BL
mTRF3-FU
mTRF4-BL
mTRF4-FU
mTRF5-BL
mTRF5-FU
mTRF6-BL
mTRF6-FU
mTRF7-BL
mTRF7-FU
mTRF8-BL
mTRF8-FU
Relative Quantity
Time

c

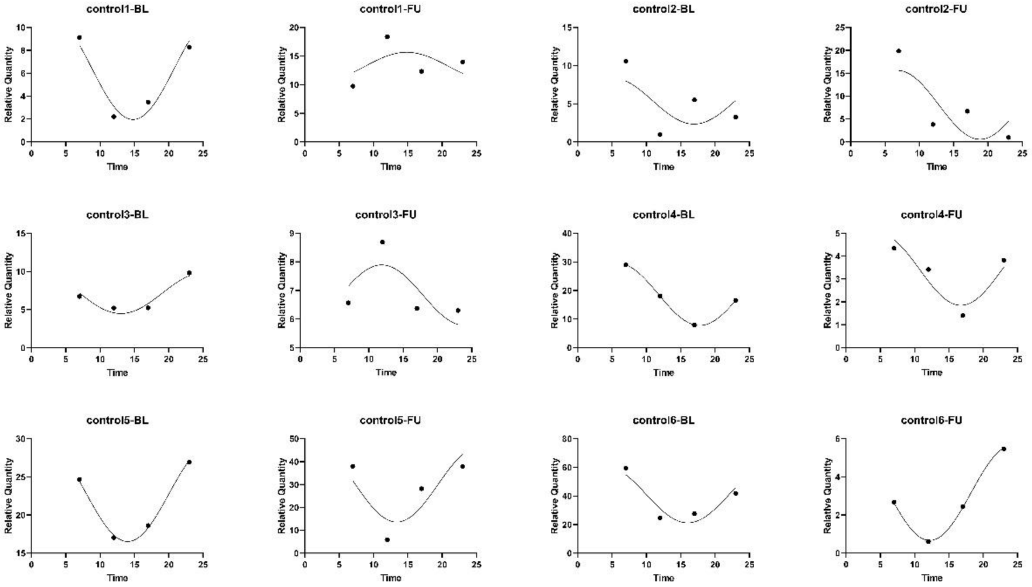
control1-BL
control1-FU
control2-BL
control2-FU
control3-BL
control3-FU
control4-BL
control4-FU
control5-BL
control5-FU
control6-BL
control6-FU
Relative Quantity
Time

d

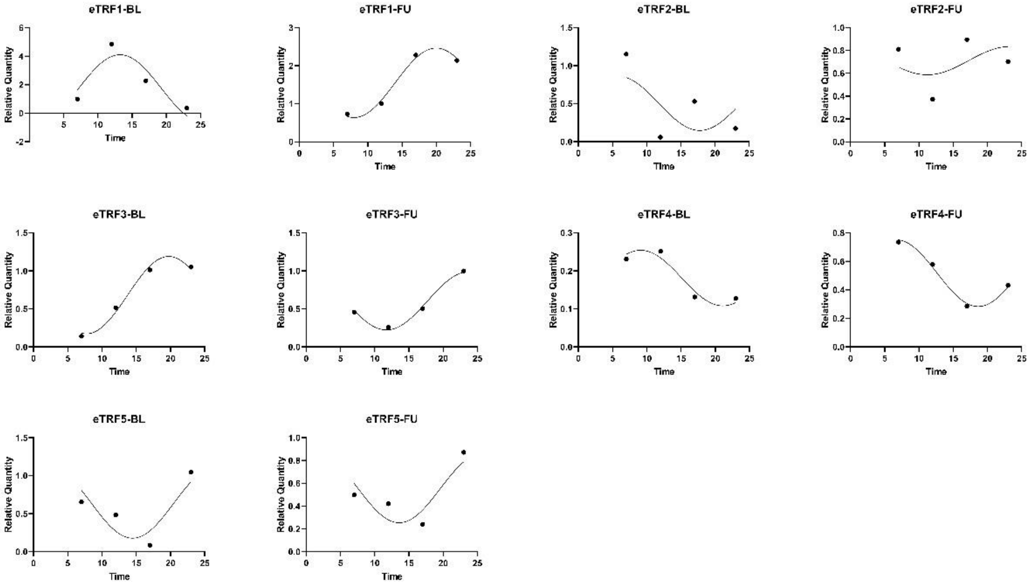
eTRF1-BL
eTRF1-FU
eTRF2-BL
eTRF2-FU
eTRF3-BL
eTRF3-FU
eTRF4-BL
eTRF4-FU
eTRF5-BL
eTRF5-FU
Relative Quantity
Time

e

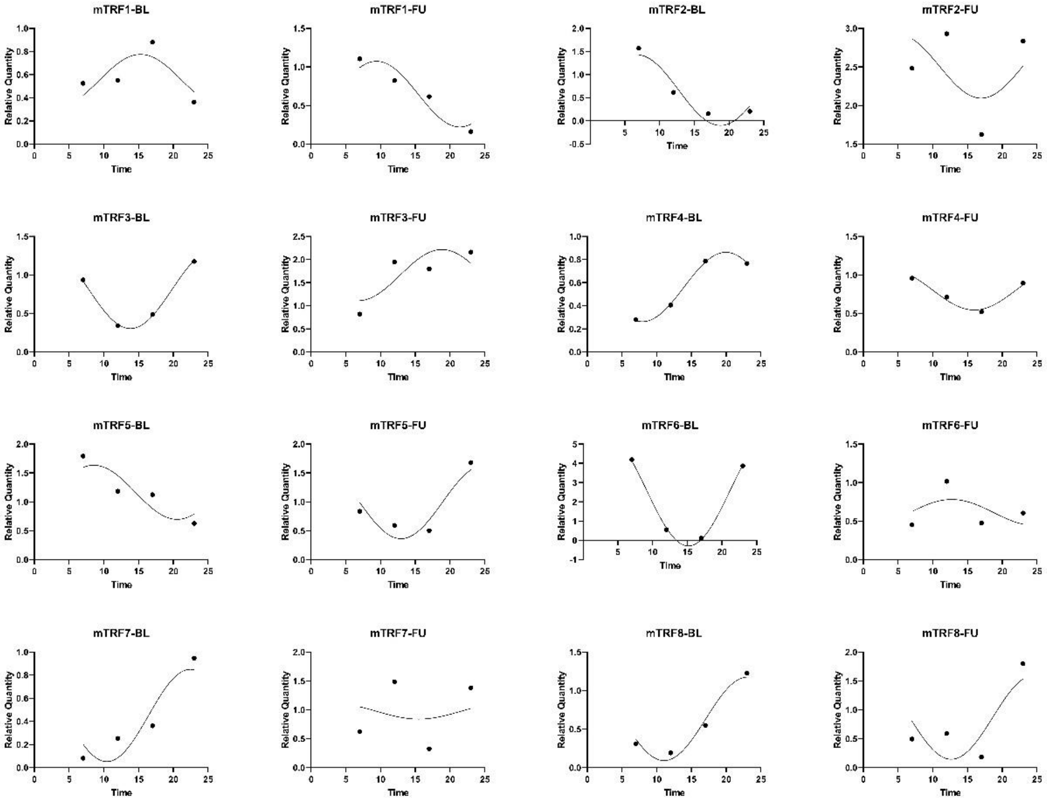
mTRF1-BL
mTRF1-FU
mTRF2-BL
mTRF2-FU
mTRF3-BL
mTRF3-FU
mTRF4-BL
mTRF4-FU
mTRF5-BL
mTRF5-FU
mTRF6-BL
mTRF6-FU
mTRF7-BL
mTRF7-FU
mTRF8-BL
mTRF8-FU
Relative Quantity
Time

f

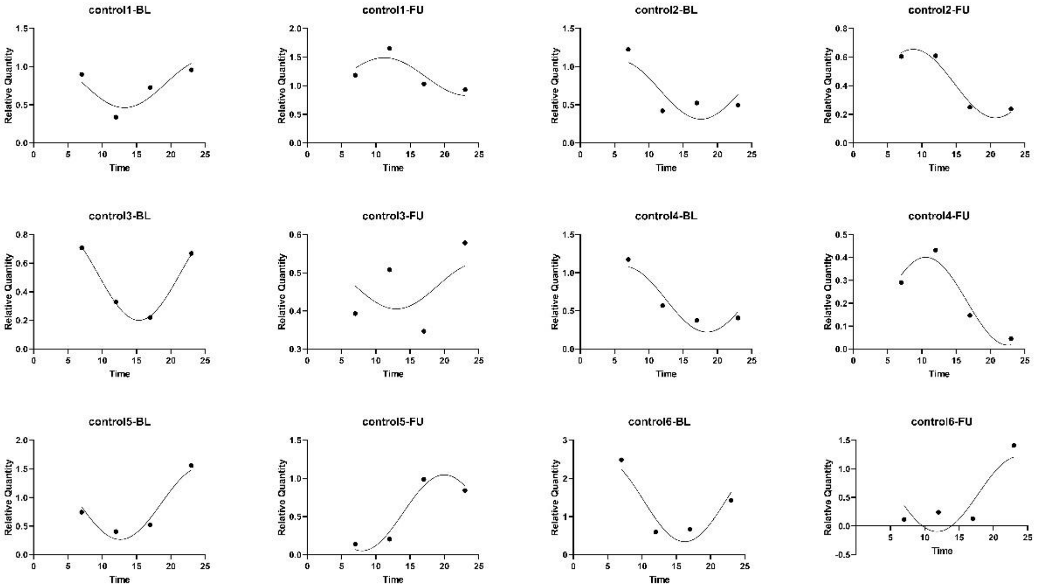
control1-BL
control1-FU
control2-BL
control2-FU
control3-BL
control3-FU
control4-BL
control4-FU
control5-BL
control5-FU
control6-BL
control6-FU
Relative Quantity
Time

g

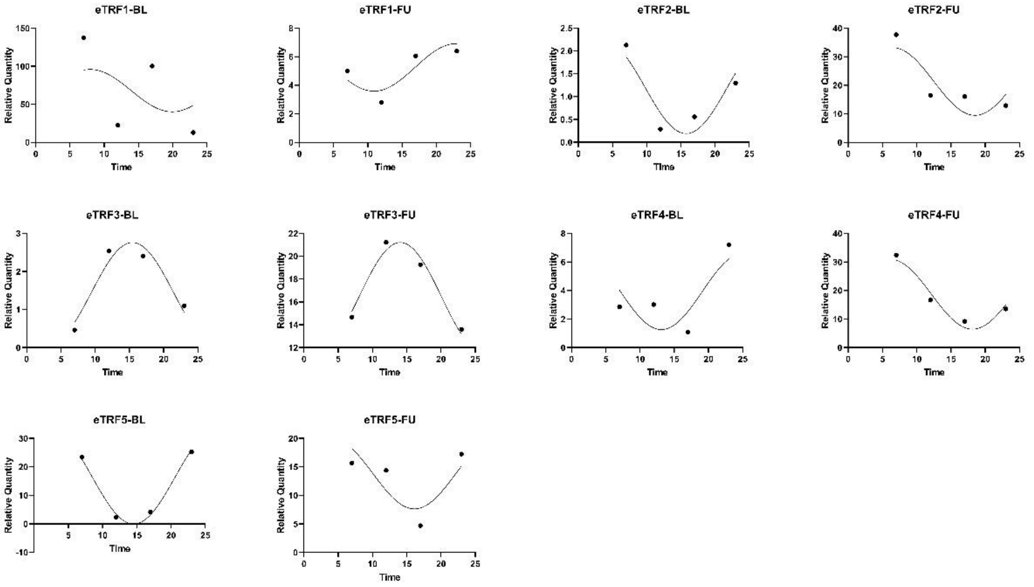
eTRF1-BL
eTRF1-FU
eTRF2-BL
eTRF2-FU
eTRF3-BL
eTRF3-FU
eTRF4-BL
eTRF4-FU
eTRF5-BL
eTRF5-FU
Relative Quantity
Time

h

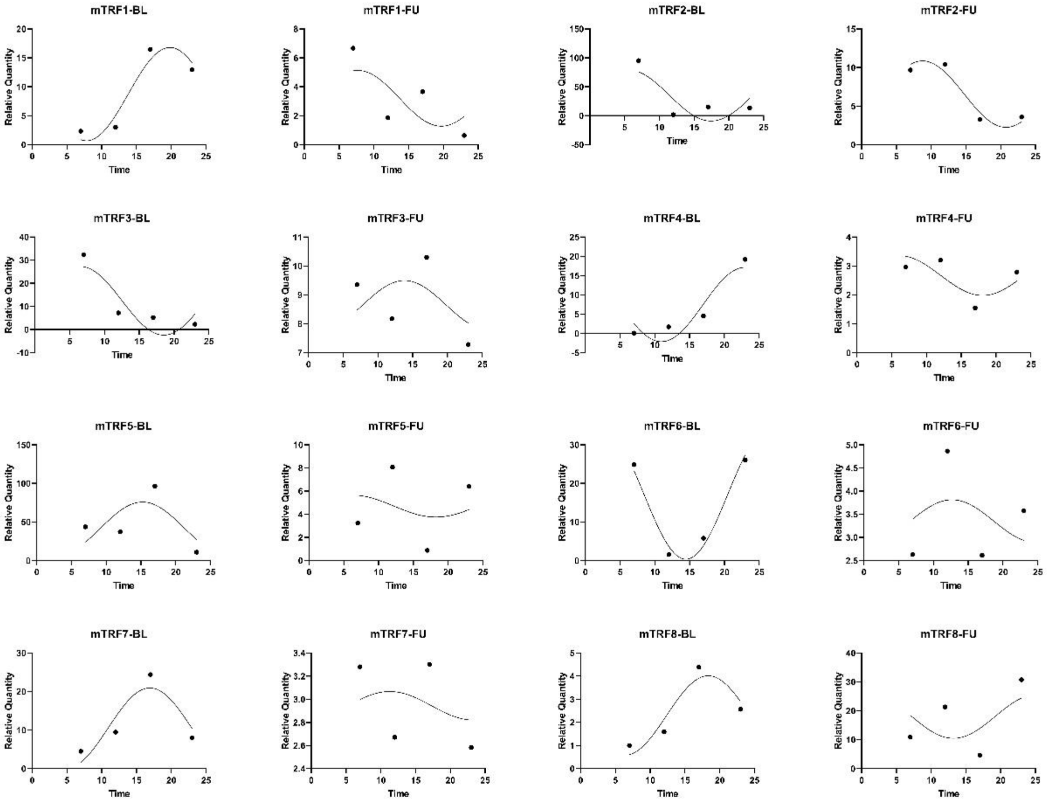
mTRF1-BL
mTRF1-FU
mTRF2-BL
mTRF2-FU
mTRF3-BL
mTRF3-FU
mTRF4-BL
mTRF4-FU
mTRF5-BL
mTRF5-FU
mTRF6-BL
mTRF6-FU
mTRF7-BL
mTRF7-FU
mTRF8-BL
mTRF8-FU
Relative Quantity
Time

i

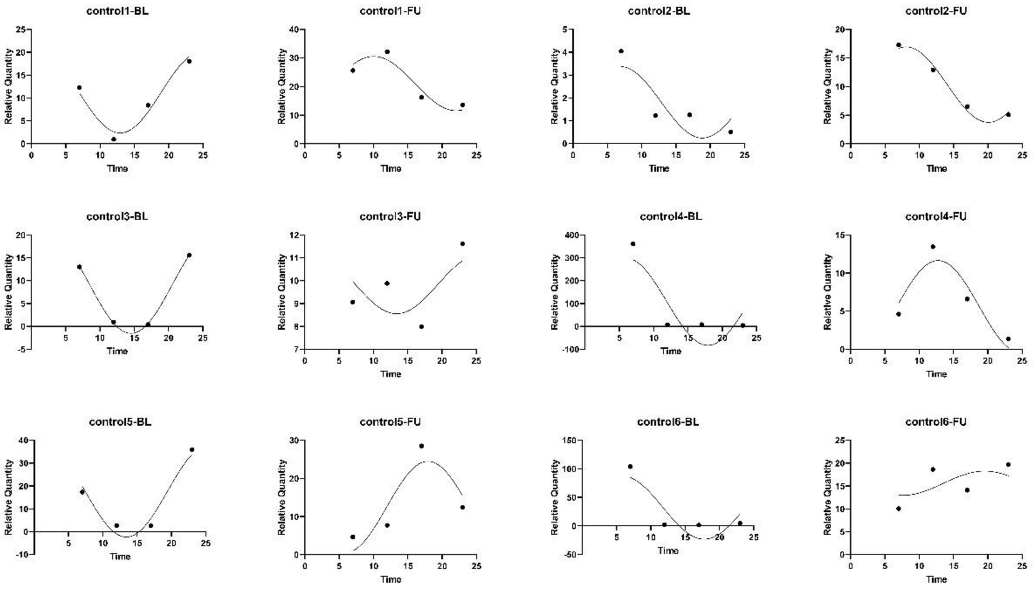

control1-BL
control1-FU
control2-BL
control2-FU
control3-BL
control3-FU
control4-BL
control4-FU
control5-BL
control5-FU
control6-BL
control6-FU
Relative Quantity
Time

j

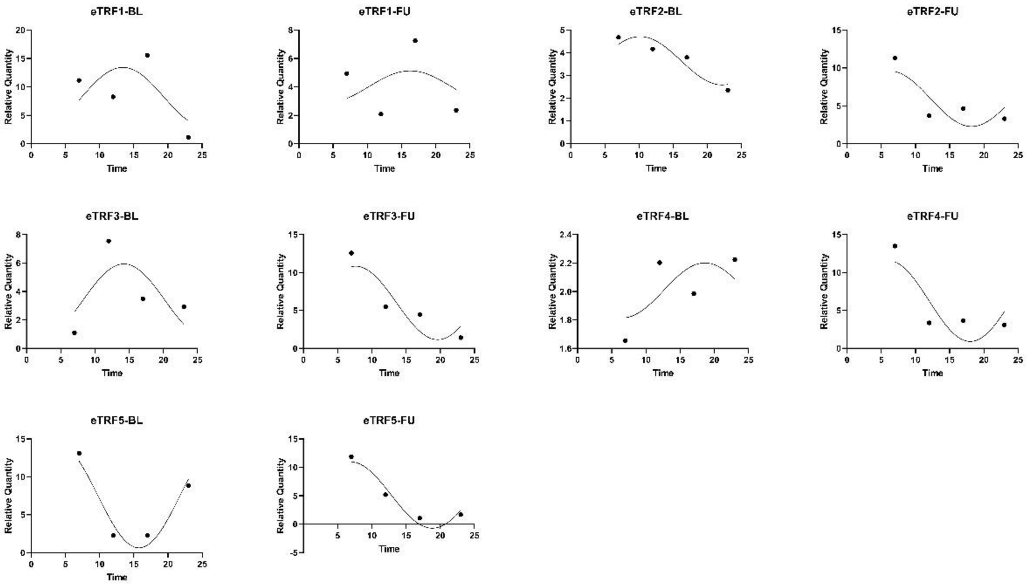

eTRF1-BL
eTRF1-FU
eTRF2-BL
eTRF2-FU
eTRF3-BL
eTRF3-FU
eTRF4-BL
eTRF4-FU
eTRF5-BL
eTRF5-FU
Relative Quantity
Time

k

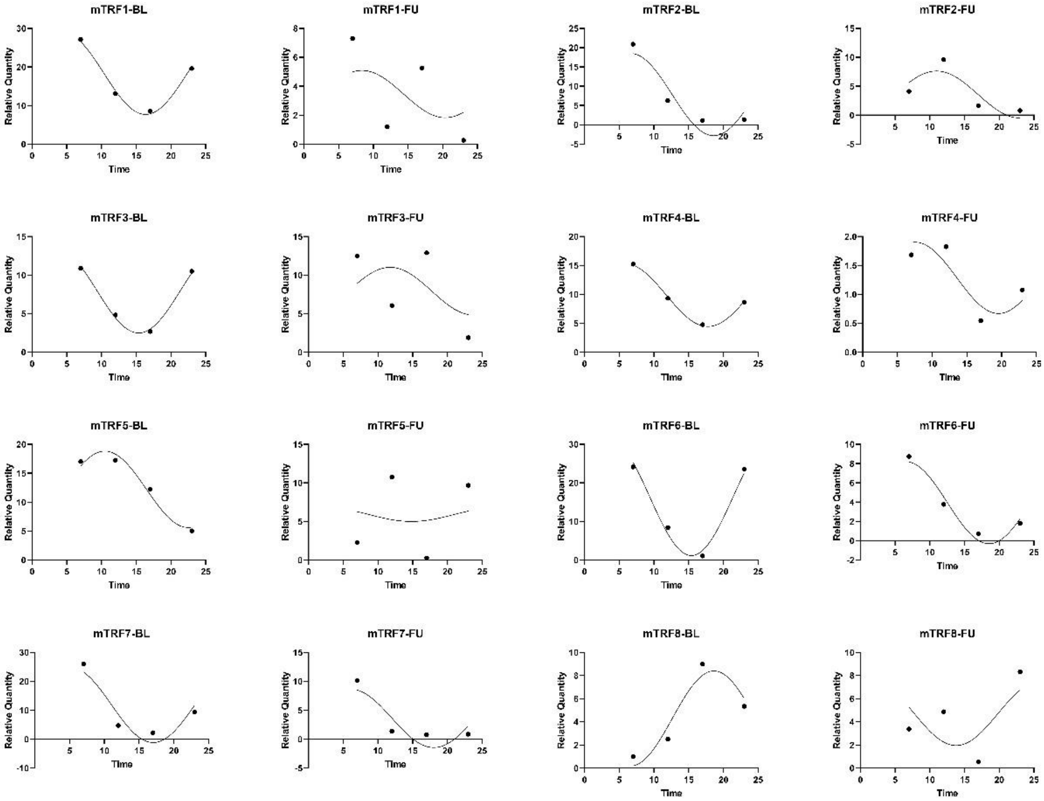
mTRF1-BL
mTRF1-FU
mTRF2-BL
mTRF2-FU
mTRF3-BL
mTRF3-FU
mTRF4-BL
mTRF4-FU
mTRF5-BL
mTRF5-FU
mTRF6-BL
mTRF6-FU
mTRF7-BL
mTRF7-FU
mTRF8-BL
mTRF8-FU
Relative Quantity
Time

l

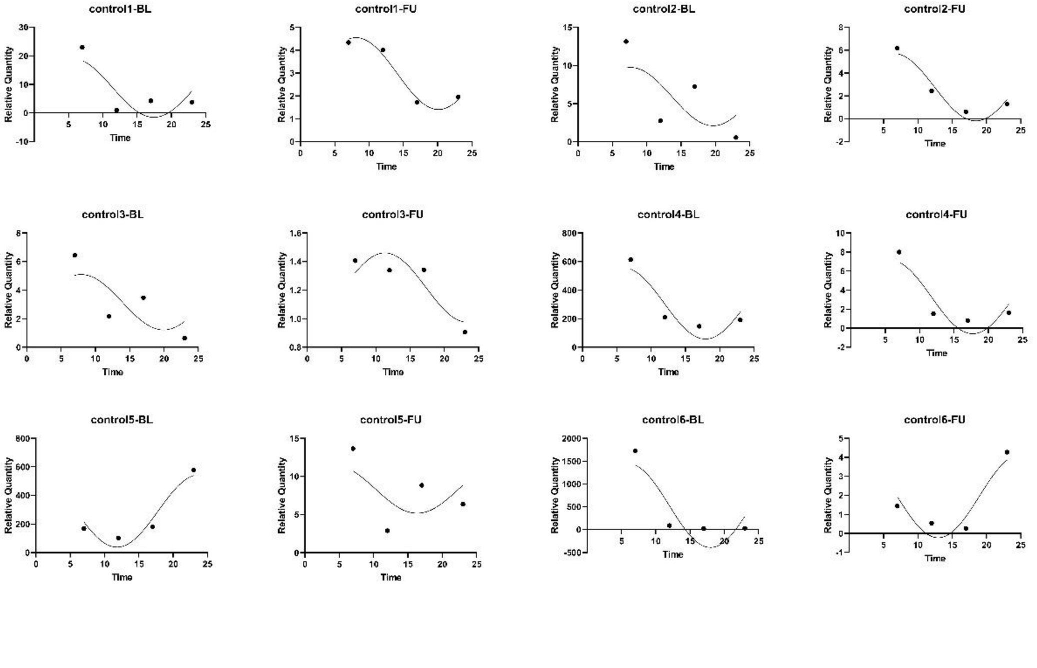
control1-BL
control1-FU
control2-BL
control2-FU
control3-BL
control3-FU
control4-BL
control4-FU
control5-BL
control5-FU
control6-BL
control6-FU
Relative Quantity
Time

m

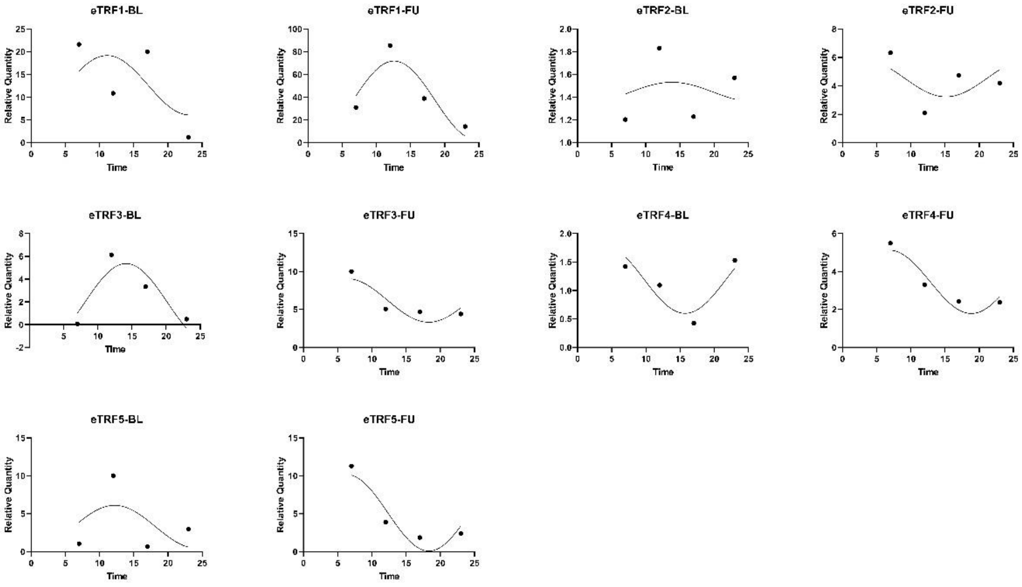
eTRF1-BL
eTRF1-FU
eTRF2-BL
eTRF2-FU
eTRF3-BL
eTRF3-FU
eTRF4-BL
eTRF4-FU
eTRF5-BL
eTRF5-FU
Relative Quantity
Time

n

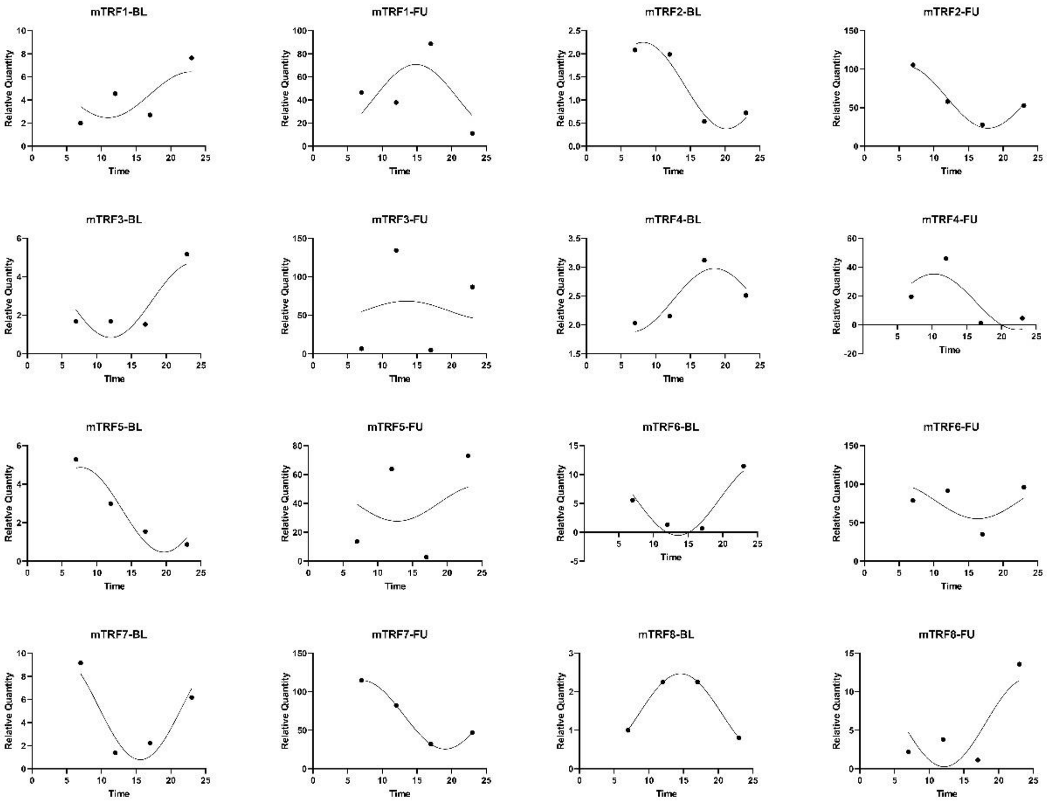
mTRF1-BL
mTRF1-FU
mTRF2-BL
mTRF2-FU
mTRF3-BL
mTRF3-FU
mTRF4-BL
mTRF4-FU
mTRF5-BL
mTRF5-FU
mTRF6-BL
mTRF6-FU
mTRF7-BL
mTRF7-FU
mTRF8-BL
mTRF8-FU
Relative Quantity
Time

o

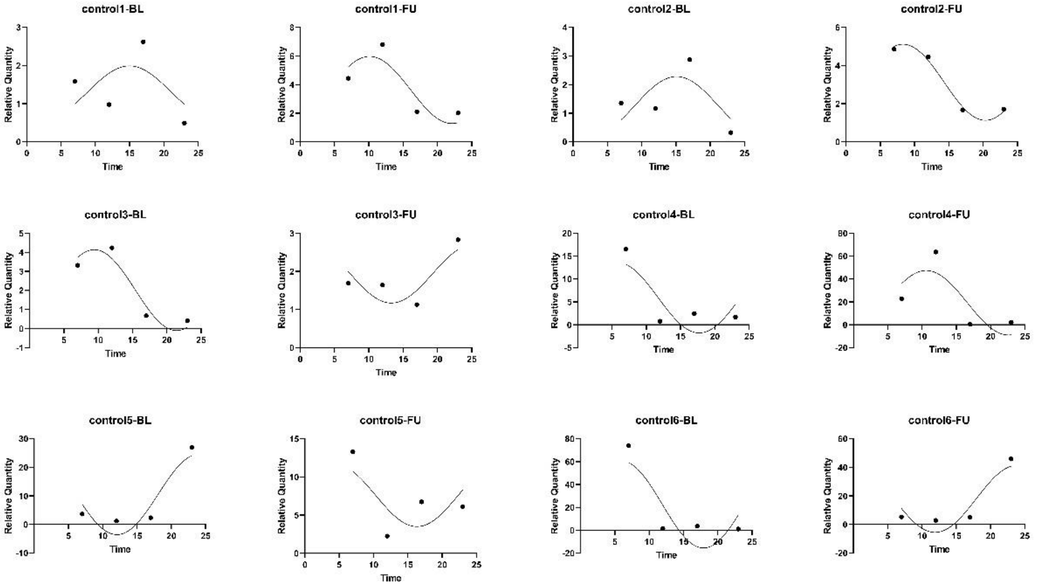
control1-BL
control1-FU
control2-BL
control2-FU
control3-BL
control3-FU
control4-BL
control4-FU
control5-BL
control5-FU
control6-BL
control6-FU
Relative Quantity
Time

p

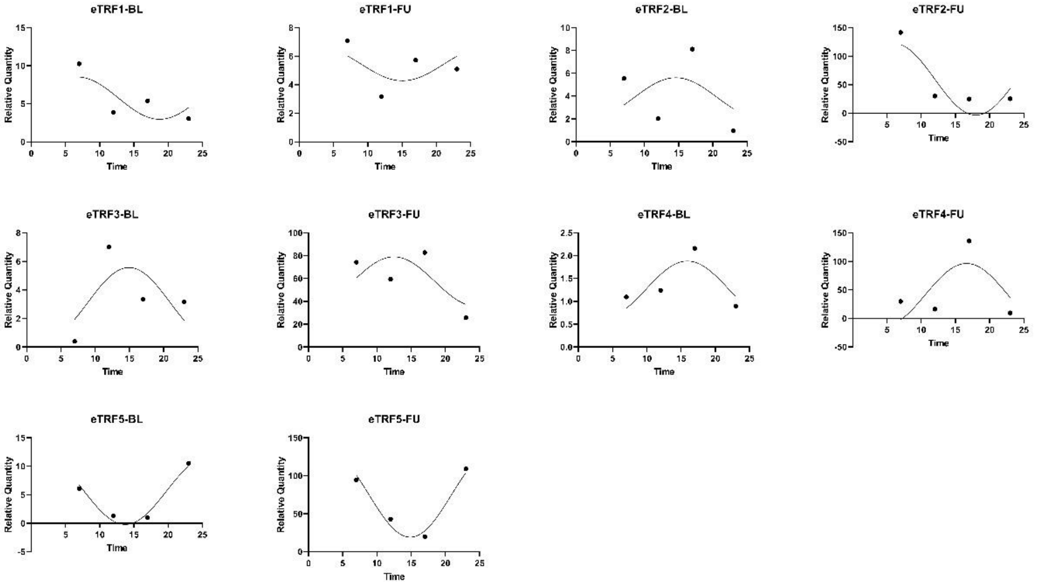
eTRF1-BL
eTRF1-FU
eTRF2-BL
eTRF2-FU
eTRF3-BL
eTRF3-FU
eTRF4-BL
eTRF4-FU
eTRF5-BL
eTRF5-FU
Relative Quantity
Time

q

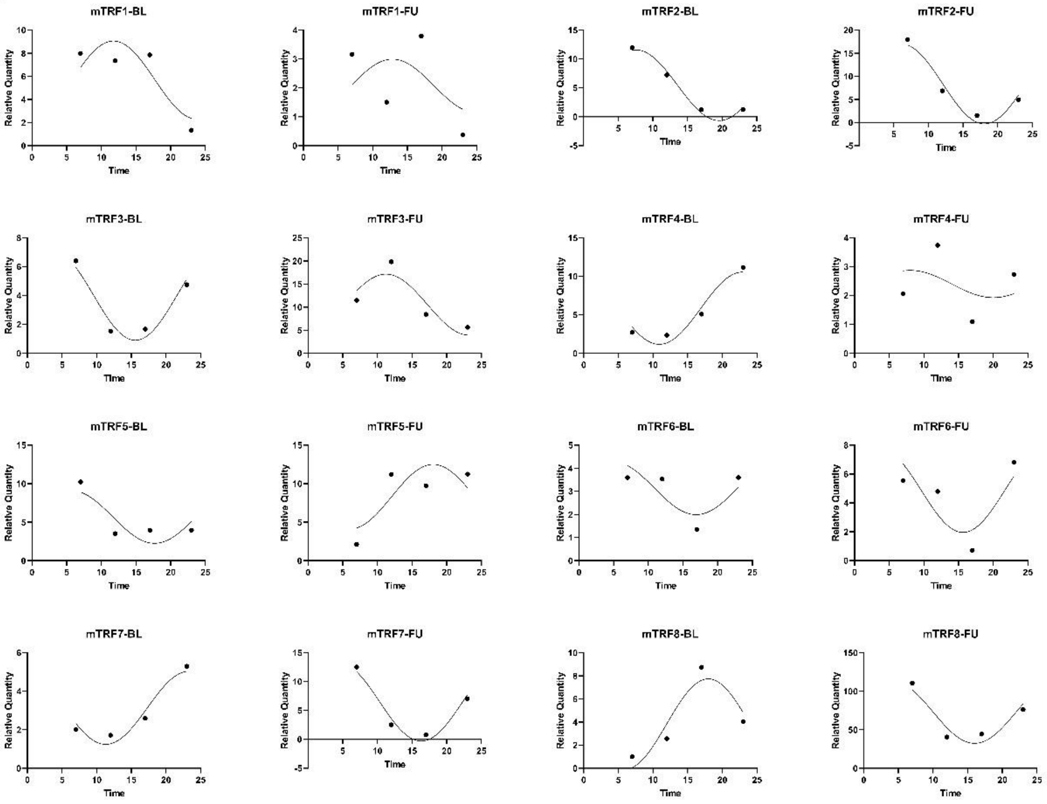
mTRF1-BL
mTRF1-FU
mTRF2-BL
mTRF2-FU
mTRF3-BL
mTRF3-FU
mTRF4-BL
mTRF4-FU
mTRF5-BL
mTRF5-FU
mTRF6-BL
mTRF6-FU
mTRF7-BL
mTRF7-FU
mTRF8-BL
mTRF8-FU
Relative Quantity
Time

r

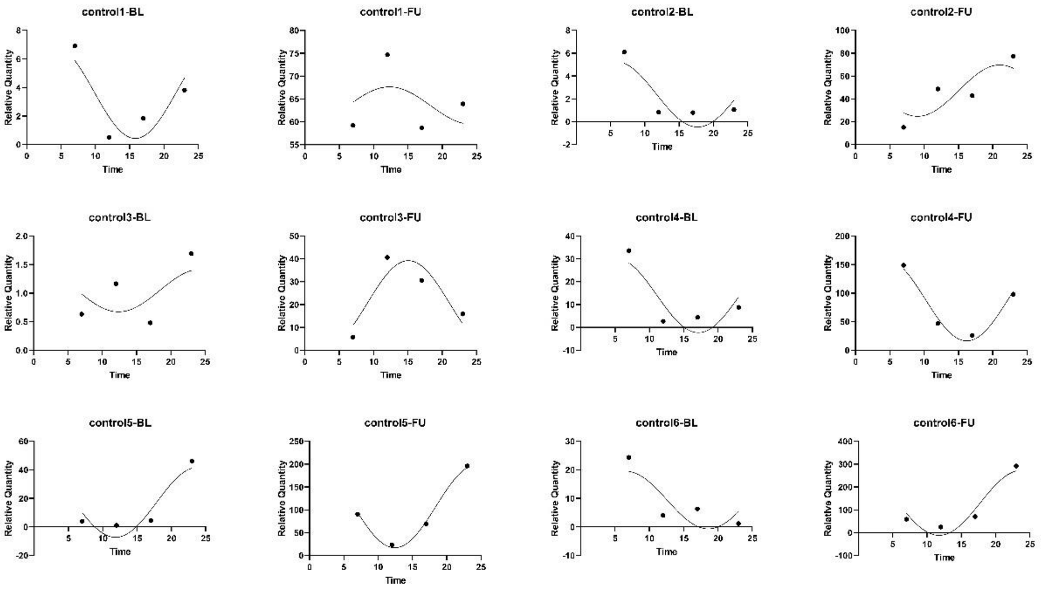
control1-BL
control1-FU
control2-BL
control2-FU
control3-BL
control3-FU
control4-BL
control4-FU
control5-BL
control5-FU
control6-BL
control6-FU
Relative Quantity
Time

s

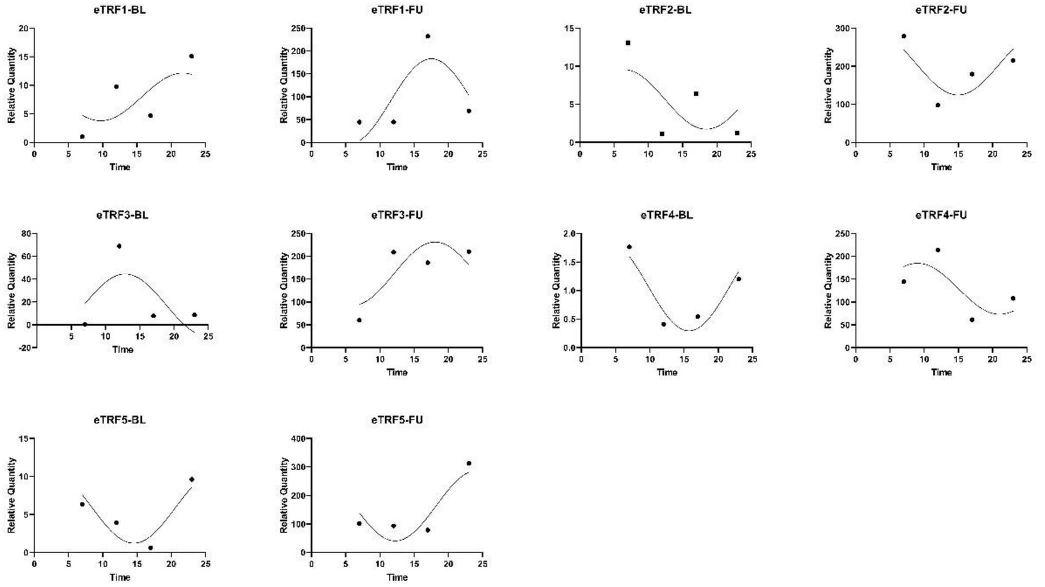
eTRF1-BL
eTRF1-FU
eTRF2-BL
eTRF2-FU
eTRF3-BL
eTRF3-FU
eTRF4-BL
eTRF4-FU
eTRF5-BL
eTRF5-FU
Relative Quantity
Time

t

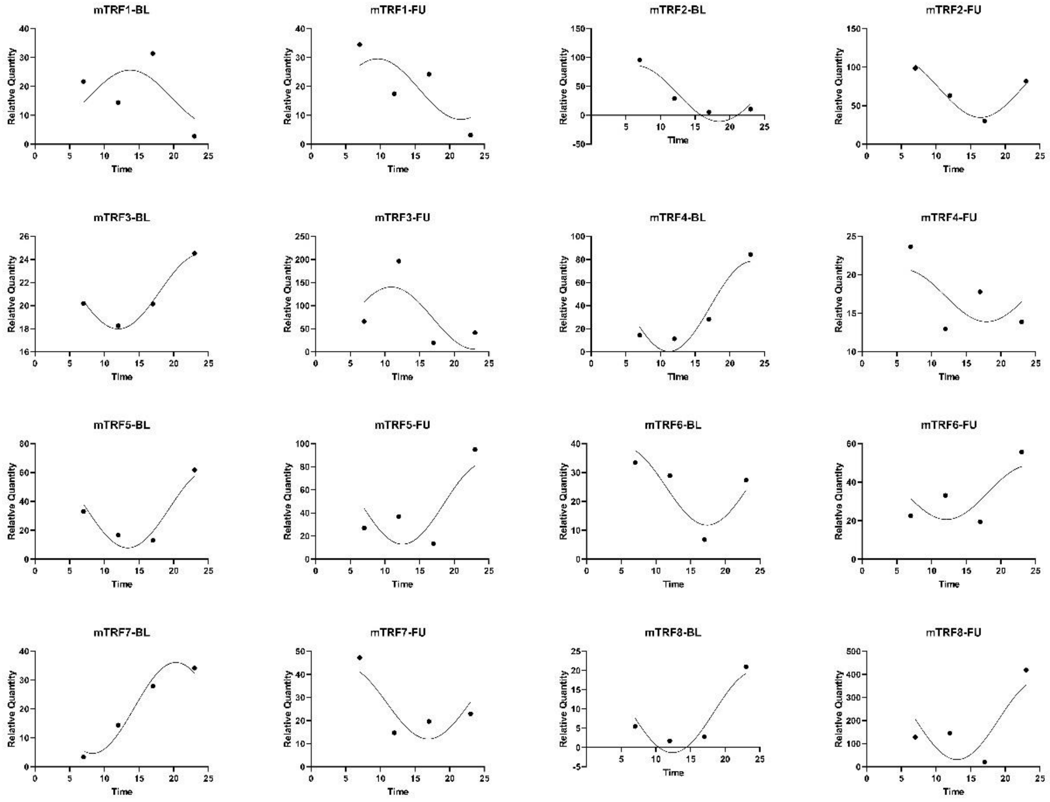
mTRF1-BL
mTRF1-FU
mTRF2-BL
mTRF2-FU
mTRF3-BL
mTRF3-FU
mTRF4-BL
mTRF4-FU
mTRF5-BL
mTRF5-FU
mTRF6-BL
mTRF6-FU
mTRF7-BL
mTRF7-FU
mTRF8-BL
mTRF8-FU
Relative Quantity
Time

u

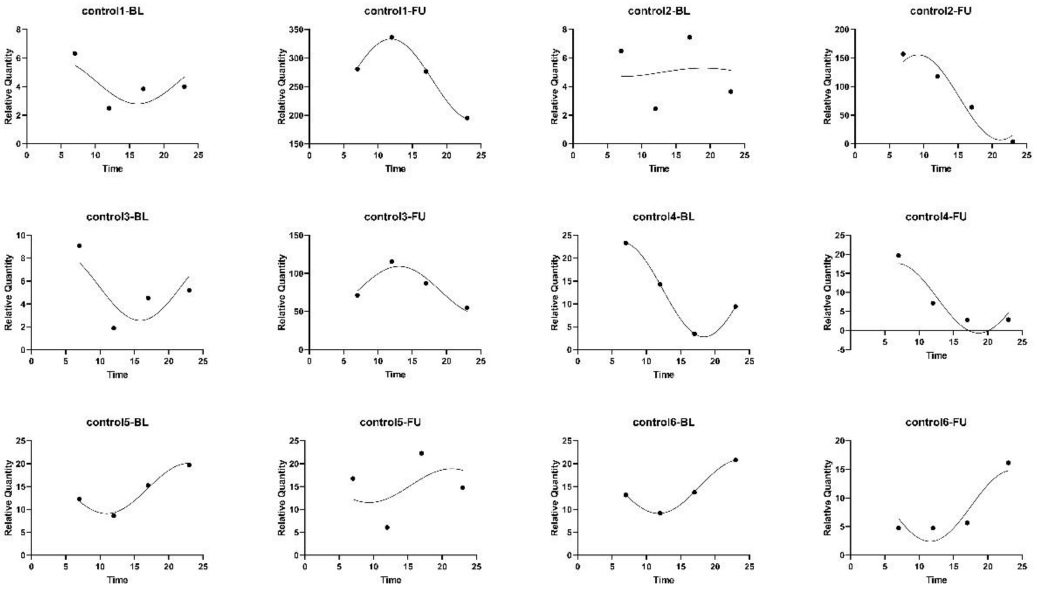

Figure S5. Cosinor models plots of clock genes mRNA levels in PBMCs of each participant who received circadian rhythm related parameters testing.

(a) Cosinor models plots of BMAL1 mRNA levels in PBMCs of each participant in eTRF group.

(b) Cosinor models plots of BMAL1 mRNA levels in PBMCs of each participant in mTRF group.

(c) Cosinor models plots of BMAL1 mRNA levels in PBMCs of each participant in control group.

(d) Cosinor models plots of CRY1 mRNA levels in PBMCs of each participant in eTRF group.

(e) Cosinor models plots of CRY1 mRNA levels in PBMCs of each participant in mTRF group.

(f) Cosinor models plots of CRY1 mRNA levels in PBMCs of each participant in control group.

(g) Cosinor models plots of CRY2 mRNA levels in PBMCs of each participant in eTRF group.

(h) Cosinor models plots of CRY2 mRNA levels in PBMCs of each participant in mTRF group.

(i) Cosinor models plots of CRY2 mRNA levels in PBMCs of each participant in control group.

(j) Cosinor models plots of PER1 mRNA levels in PBMCs of each participant in eTRF group.

(k) Cosinor models plots of PER1 mRNA levels in PBMCs of each participant in mTRF group.

(l) Cosinor models plots of PER1 mRNA levels in PBMCs of each participant in control group.

(m) Cosinor models plots of PER2 mRNA levels in PBMCs of each participant in eTRF group.

(n) Cosinor models plots of PER2 mRNA levels in PBMCs of each participant in mTRF group.

(o) Cosinor models plots of PER2 mRNA levels in PBMCs of each participant in control group.

(p) Cosinor models plots of PER3 mRNA levels in PBMCs of each participant in eTRF group.

(q) Cosinor models plots of PER3 mRNA levels in PBMCs of each participant in mTRF group.

(r) Cosinor models plots of PER3 mRNA levels in PBMCs of each participant in control group.

(s) Cosinor models plots of SIRT1 mRNA levels in PBMCs of each participant in eTRF group.

(t) Cosinor models plots of SIRT1 mRNA levels in PBMCs of each participant in mTRF group.

(u) Cosinor models plots of SIRT1 mRNA levels in PBMCs of each participant in control group.

BL: baseline; FU: follow-up.

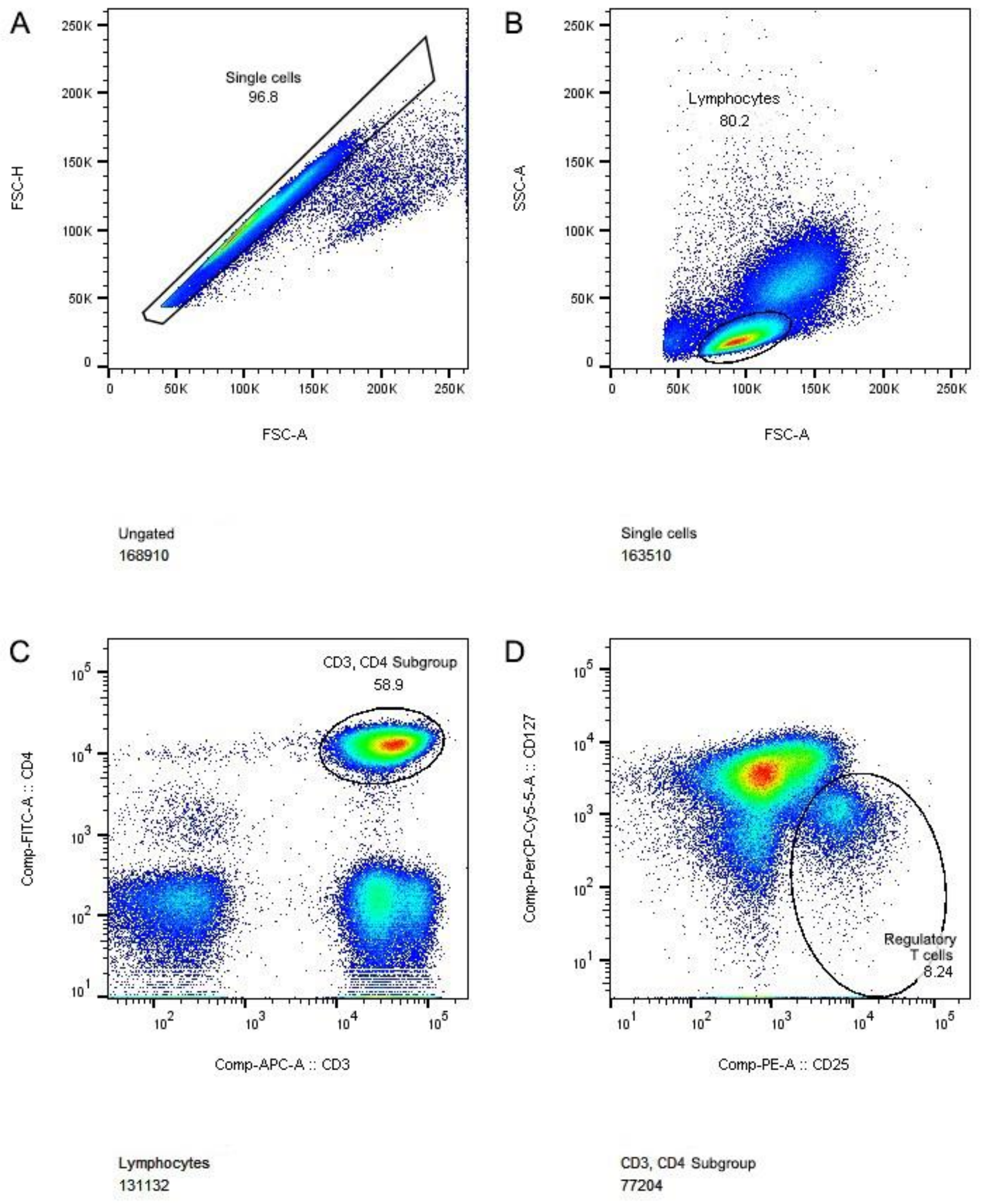

Figure S6. Gating strategy of flow cytometry.

Each pseudocolor plot represents a cell analyzed by flow cytometry and the axis labels represent the marker and fluorochrome we used in the analysis. All four figures (A-D) descript the gating strategy.

(A) According to the physical characteristics of flow cytometry, FSC-A/FSC-H was used to distinguish adhesive cells, and located single cells on the diagonal;

(B) FSC-A/SSC-A was used to circle the lymphocytes group of which the FSC-A signal is larger than fragments and smaller than granulocytes, and the SSC-A signal is smaller than granulocytes;

(C) CD3 APC/CD4 FITC were used to circle CD4+ T lymphocyte;

(D) According to the biological characteristics of regulatory T cells (Tregs), in the CD4+ T lymphocyte population, Tregs were delineated by CD127low/CD25+.

Protocols for "Randomized controlled trial for time-restricted eating in healthy volunteers without obesity"

Study design

Study design

We conducted a randomized, controlled trial, in which participants were randomized to an eTRF group (eating during no more than 8 h between 06:00 and 15:00 and fasting for the rest of the day), an mTRF group (eating during no more than eight hours between 11:00 and 20:00 and fasting for the rest of the day), and a control group (eating *ad libitum* over 8 h each day). Participants in the eTRF and mTRF groups were only allowed to consume water, flavored carbonated water, unsweetened tea, and coffee during the fasting period. The primary outcome was the change in HOMA-IR, an index of insulin resistance that is calculated using the fasting glucose and insulin concentrations. The secondary outcomes were changes in energy intake, fasting glucose, body mass, body composition, blood pressure, blood lipid concentrations, inflammatory markers, liver enzymes, immune cells, gut microbiota, sleep quality and appetite. Change in daily rhythms of plasma adipokine concentrations and PBMC clock gene expression were measured as exploratory analyses.

Study participants

This clinical trial was conducted at Peking Union Medical College Hospital (PUMCH, China), approved by the hospital's ethics committee, and conducted according to the Helsinki Declaration of 1975. Prior to enrolling participants, the study was registered

at chictr.org.cn (ChiCTR2000029797). Participants were recruited from the Beijing area from Feb. 16th, 2020, to Mar. 22nd, 2020, by means of posters, emails, flyers, social media, and website advertisements. Ninety participants who were in the habit of eating over more than 8 hours per day and who did not have recent experience of fasting were recruited into the trial after providing their written informed consent.

Diets and compliance

Participants in the eTRF group were instructed to choose an 8-hour eating period between 6:00 and 15:00 and to fast for the rest of the day. Those in mTRF group were instructed to choose an 8-hour eating period between 11:00 and 20:00 and to fast for the rest of the day. Participants in the control group could eat *ad libitum*, following their usual eating regimens, with food being consumed over more than 8 h per day. The participants maintained their habitual alcohol intake during the trial, which was no more than twice a week, as required in the eligibility criteria. Alcohol intake was forbidden on the test days and the preceding days. To ensure compliance, participants were required to take photos of their food as they began to eat and as they finished and to send them privately to the investigators using a WeChat-supported web message-sending applet. All participants wrote a consent form and guaranteed to supply real data about food intake at the beginning of the trial. The investigators checked their posts every day and participants who failed to post those photos for more than 3 days, which meant they could not fulfill the required 90% completion rate, were considered to have failed to complete the trial. The energy content of each meal was estimated using China

Food Composition Database. One designated researcher who had got a good clinical practice certificate was trained to estimate the number of different types of food using the posted photos, which would be double-checked by another researcher. Standardized measurement guides were used to assess portion sizes. The records for all the meals of every participant were included in the analysis, except for non-compliant days. To estimate compliance, the number of person-days for each group was defined as 35 days (the length of the trial) multiplied by the number of participants who finished the trial. The compliance rate was calculated as the number of self-reported compliant days divided by the total number of person-days for each group. Because 28, 26, and 28 participants in the eTRF, mTRF, and control groups, respectively, completed the trial, the compliance levels were calculated to be 980 (35 × 28) for the eTRF group and 910 (35 × 26) for the mTRF group. Because participants were instructed to take either TRF regimen or normal diet regimen, they were not blinded to the assignment of the groups. Investigators who checked posted photos and estimated energy contents from photos were not blinded to the assignment of the group. Other investigators and statisticians were blinded during the study procedure, and were unblinded after all the data had been analyzed.

Randomization procedure

For the pilot RCT, participants were randomly assigned to either the eTRF, mTRF, or control group in a 1:1:1 ratio, using a computer-based random-number generator by designated researchers.

Inclusion and exclusion criteria

The inclusion criteria were: 1) 18–64 years old; 2) ability to attend the hospital at regular intervals; 3) ability to independently provide informed consent; 4) BMI between 17.5 and 30.0 kg/m^2; 5) daily feeding period of more than 8 hours; and 6) stable body mass (change <±10% of current body mass during the 3 months prior to the study). The exclusion criteria were: 1) night-shift work more than once a week; 2) fasting during the preceding 8 weeks; 3) alcohol consumption more than twice a week; 4) pregnancy, gastrointestinal abnormalities or eating disorders, history of gastrointestinal surgery or systemic disease; 5) use of corticosteroid drugs, β-receptor blockers, or other drugs that might affect the findings; 6) a diagnosis of hypertension, diabetes, or other metabolic disease; and 7) a diagnosis of insomnia.

Anthropometric measurements

Body mass and percentage body fat were measured using an HBF-371 Bioelectrical impedance analyzer (Omron Healthcare Co., Kyoto, Japan). Height was measured using a metric tape, with the participant standing up straight against a wall. BMI was calculated using the body mass in kilograms divided by the height in meters, squared.

Blood sampling and storage

Blood sampling was performed at the beginning and the end of the trial. Fasting blood sampled were collected during the morning (07:00–08:30) after an overnight fast of at

least 8 hours. For those who participated in the analysis of daily rhythms, blood sampling was performed at 07:00, after an overnight fast, and at 12:00, 17:00, and 23:00. Plasma, serum, whole blood, and PBMC fractions were collected and either analyzed immediately or stored at −80°C until assayed.

Flow cytometric analysis

PBMCs were separated from blood samples using Ficoll (GE Healthcare, Chicago, IL) and centrifugation. pTregs were counted by flow cytometry (FACS Canto plus, BD Biosciences, Franklin Lakes, NJ) using a fixed staining protocol of 5µl antibody (Anti-human CD4 (RPA-T4) FITC, 11-0049-41; Anti-human CD25 (BC96) PE, 12-0259-41; Anti-human CD3 (UCHT1) APC, 17-0038-41; Anti-human CD127 (EBIORDR5), PERCP-CYAN, 45-1278-41; eBioscience, San Diego, CA) diluted in 100µl PBS. Flow cytometric data were analyzed using FlowJo (Version 10.6.2, BD Biosciences) (Figure S6).

Fecal sample collection and storage

Fecal samples were collected during the 3 days before the start of the trial and during the same period of time before the end of the trial. Detailed instructions regarding sample collection and transportation were provided by the study personnel and the participants were provided with containers with feces-preserving fluid. The participants were asked to collect approximately 2–3 g of feces using the spatula attached to the cover of the container, to place the fecal sample inside, and then to shake the container

well. The containers were then delivered to investigators within 24 h and stored at −80°C until the contents were analyzed.

Biochemical measurements

The plasma activities of AST, ALT, ALP, GGT, and lactate dehydrogenase; and the concentrations of LDL-C, HDL-C, total cholesterol, triglyceride, and glucose were measured using an automated analyzer (Beckmann-Coulter AU 5800, Brea, CA). Insulin was measured using an ADVIA Centaur XP (Siemens, Munich, Germany). Blood cells were analyzed using XN-2000 (Sysmex, Kobe, Japan). The IL-8 and TNF-α concentrations were measured using an Immulite 1000 (Siemens). The concentrations of resistin (AdipoGen Life Science, Liestal, Switzerland), leptin (Phoenix Pharmaceuticals, Burlingame, CA), and ghrelin (Thermo Fisher, Waltham, MA) were measured using ELISA kits on a microplate reader (Bio-Rad Laboratories).

Real-time quantitative PCR

RNA was pooled from PBMCs and used for cDNA synthesis. Transcript levels were then quantified by qPCR using SYBR qPCR mix (ABI-Invitrogen). The expression of each gene of interest was normalized to that of *ACTB* using the $2^{-\triangle\triangle CT}$ method. The primer sequences are listed in Table S3.

Subjective sleep quality and eating habits

The participants were required to maintain their normal sleeping habits throughout the trial and to avoid undergoing testing after a night shift. Sleep was analyzed using the PSQI questionnaire and eating habits were analyzed using the CNAQ.

Analysis of the microbiota

DNA was obtained from fecal samples using the QIAamp Fast DNA Stool Mini Kit (Qiagen), according to the manufacturer's protocol. The concentrations of the extracted DNA were measured using a Nanodrop, and DNA electrophoresis using a 1% agarose gel was performed to verify its integrity. To generate the 16S rDNA library, PCR analysis was performed using a 16S V3-V4 hypervariable region general primer set and a KAPA HiFi Hotstart ReadyMix PCR Kit (KAPA), and the PCR products were collected using an AxyPrep DNA gel extraction kit (Axygen). To establish qualified 16S rDNA libraries, the concentrations were measured using the Nanodrop, 1% agarose gel electrophoresis was performed, and quantitative testing with Qubit dsDNA HS Assay Kit was performed prior to sequencing. The 16S rDNA amplicon sequence results were analyzed using the Hiseq 2500 PE250 platform. The sequencing results were first assembled using PANDAseq 2.11 software to acquire clean reads. An operational taxonomic units table was constructed using Usearch 10.0.259 and randomized leveling was performed on each sample to avoid sample size-related bias. Alpha diversity, assessed using chao1, was analyzed using QIIME 2 2017.6.0, and the chao1 changes during the study were evaluated using one-way repeated-measures ANOVA, followed by the Holm-Sidak multiple comparisons test. Analyses of the

changes in the gut microbial profiles were performed using LEfSe 1.0. PICRUSt 1.0.0, based on closed-reference operational taxonomic units, was used to predict the abundances of functional categories, on the basis of COG analysis.
