# Supplementary material for: Randomized controlled trial for time-restricted eating in healthy volunteers without obesity
Source: Nat Commun. 2022 Feb 22;13:1003. doi: 10.1038/s41467-022-28662-5 (PMC8864028; doi:10.1038/s41467-022-28662-5)
Supplement: Supplementary file 3 — Reporting Summary [file 41467_2022_28662_MOESM3_ESM.pdf]

nature research

Corresponding author(s): Yilei Mao, Huayu Yang

Last updated by author(s): Dec 8, 2021

Reporting Summary

Nature Research wishes to improve the reproducibility of the work that we publish. This form provides structure for consistency and transparency in reporting. For further information on Nature Research policies, see our Editorial Policies and the Editorial Policy Checklist.

Statistics

For all statistical analyses, confirm that the following items are present in the figure legend, table legend, main text, or Methods section.

n/a	Confirmed	
☐	☒	The exact sample size (*n*) for each experimental group/condition, given as a discrete number and unit of measurement
☐	☒	A statement on whether measurements were taken from distinct samples or whether the same sample was measured repeatedly
☐	☒	The statistical test(s) used AND whether they are one- or two-sided<br>*Only common tests should be described solely by name; describe more complex techniques in the Methods section.*
☐	☒	A description of all covariates tested
☐	☒	A description of any assumptions or corrections, such as tests of normality and adjustment for multiple comparisons
☐	☒	A full description of the statistical parameters including central tendency (e.g. means) or other basic estimates (e.g. regression coefficient) AND variation (e.g. standard deviation) or associated estimates of uncertainty (e.g. confidence intervals)
☐	☒	For null hypothesis testing, the test statistic (e.g. *F*, *t*, *r*) with confidence intervals, effect sizes, degrees of freedom and *P* value noted<br>*Give P values as exact values whenever suitable.*
☒	☐	For Bayesian analysis, information on the choice of priors and Markov chain Monte Carlo settings
☐	☒	For hierarchical and complex designs, identification of the appropriate level for tests and full reporting of outcomes
☒	☐	Estimates of effect sizes (e.g. Cohen's *d*, Pearson's *r*), indicating how they were calculated

Our web collection on statistics for biologists contains articles on many of the points above.

Software and code

Policy information about availability of computer code

Data collection	Microsoft Excel (Microsoft 365 MSO version 1904)
Data analysis	Software: Graphpad Prism 7.0 (Graphpad software, La Jolla, Calif., USA), FlowJo (Version 10.6.2, BD Biosciences) , PANDAseq 2.11, Usearch 10.0.259, QIIME 2 2017.6.0, LEfSe 1.0, PICRUSt 1.0.0<br>Equation for Cosinor analysis using non-linear regression in Graphpad Prism: Y=Baseline+Amplitude*cos(2*3.14*X/24+Phaseshift)

For manuscripts utilizing custom algorithms or software that are central to the research but not yet described in published literature, software must be made available to editors and reviewers. We strongly encourage code deposition in a community repository (e.g. GitHub). See the Nature Research guidelines for submitting code & software for further information.

Data

Policy information about availability of data

All manuscripts must include a data availability statement. This statement should provide the following information, where applicable:

- Accession codes, unique identifiers, or web links for publicly available datasets
- A list of figures that have associated raw data
- A description of any restrictions on data availability

The metagenomic sequencing data of 164 faecal samples in this study can be accessed from the BioProject Database of National Centre for Biotechnology Information with the dataset accession number PRJNA786689. The source data underlying Fig. 4, and Supplementary Figs. 1, 2 are provided as a source data file. Further information and requests for resources and data should be directed to and will be fulfilled by the corresponding author upon reasonable request. The study was approved by Chinese Ministry of Science and Technology (MOST) for the Review and Approval of Human Genetic Resources (approval number 2021BAT2235). China Food Composition Database was used in this manuscript 74.

Field-specific reporting

Please select the one below that is the best fit for your research. If you are not sure, read the appropriate sections before making your selection.

- [x] Life sciences
- [ ] Behavioural & social sciences
- [ ] Ecological, evolutionary & environmental sciences

For a reference copy of the document with all sections, see nature.com/documents/nr-reporting-summary-flat.pdf

Life sciences study design

All studies must disclose on these points even when the disclosure is negative.

Sample size	For the sample size calculation, we estimated that the eTRF group would show a 37.5% reduction in HOMA-IR and that the mTRF group would show no change in HOMA-IR during the 5-week trial. The reported mean and standard deviation are 1.91 and 0.8 in non-obese Chinese people. Therefore, to detect a 37.5% difference in HOMA-IR (1.91 × 0.375 = 0.7) between the TRF groups, the statistical power analysis indicated that 22 participants would have to complete the trial in each group in order to achieve 80% power (two-sided test, a = 0.05), assuming a within-participants standard deviation of 0.8. Taking into consideration potential drop-outs during the trial, 30 participants were recruited for each group.
Data exclusions	Eight participants were excluded from data analysis for not completing the trial
Replication	In this trial, a total of 429 participants were screened for eligibility, of whom 90 participants underwent randomization: 30 were randomly assigned to the eTRF group, 30 to the mTRF group, 30 to the control group.
Randomization	For the randomized controlled trial, participants were randomly assigned to either eTRF, mTRF or control group, in a 1:1:1 ratio, using a computer-based random-number generator.
Blinding	Because participants were instructed to take either TRF regimen or normal diet regimen, they were not blinded to the assignment of the groups. Investigators who checked posted photos and estimated energy contents from photos were not blinded to the assignment of the group. Other investigators and statisticians were blinded during the study procedure, and were unblinded after all the data had been analyzed.

Reporting for specific materials, systems and methods

We require information from authors about some types of materials, experimental systems and methods used in many studies. Here, indicate whether each material, system or method listed is relevant to your study. If you are not sure if a list item applies to your research, read the appropriate section before selecting a response.

Materials & experimental systems

n/a	Involved in the study	
[ ]	[x]	Antibodies
[x]	[ ]	Eukaryotic cell lines
[x]	[ ]	Palaeontology and archaeology
[x]	[ ]	Animals and other organisms
[ ]	[x]	Human research participants
[ ]	[x]	Clinical data
[x]	[ ]	Dual use research of concern

Methods

n/a	Involved in the study	
[x]	[ ]	ChIP-seq
[ ]	[x]	Flow cytometry
[x]	[ ]	MRI-based neuroimaging

Antibodies

Antibodies used	Anti-human CD4 (RPA-T4) FITC eBioscience 11-0049-41<br>Anti-human CD25 (BC96) FITC eBioscience 12-0259-41<br>Anti-human CD3 (UCHT1) APC eBioscience 17-0038-41<br>Anti-human CD127 (EBIORDR5) PERCP-CYAN eBioscience 45-1278-41
Validation	Anti-human CD4 (RPA-T4) FITC eBioscience 11-0049-41: Lecciso M, Ocadlikova D, et al. ATP Release from Chemotherapy-Treated Dying Leukemia Cells Elicits an Immune Suppressive Effect by Increasing Regulatory T Cells and Tolerogenic Dendritic Cells. Front Immunol. 2017 Dec 22;8:1918. doi: 10.3389/fimmu.2017.01918. PMID: 29312358; PMCID: PMC5744438.<br>Anti-human CD25 (BC96) FITC eBioscience 12-0259-41: Schaue D, et al. T-cell responses to survivin in cancer patients undergoing radiation therapy. Clin Cancer Res. 2008 Aug 1;14(15):4883-90. doi: 10.1158/1078-0432.CCR-07-4462. PMID: 18676762; PMCID: PMC2748652.<br>Anti-human CD3 (UCHT1) APC eBioscience 17-0038-41: Lecciso M, et al. ATP Release from Chemotherapy-Treated Dying Leukemia Cells Elicits an Immune Suppressive Effect by Increasing Regulatory T Cells and Tolerogenic Dendritic Cells. Front Immunol. 2017 Dec 22;8:1918. doi: 10.3389/fimmu.2017.01918. PMID: 29312358; PMCID: PMC5744438.<br>Anti-human CD127 (EBIORDR5) PERCP-CYAN eBioscience 45-1278-41: Su S, et al Blocking the recruitment of naive CD4+ T cells

reverses immunosuppression in breast cancer. Cell Res. 2017 Apr;27(4):461-482. doi: 10.1038/cr.2017.34. Epub 2017 Mar 14. PMID: 28290464; PMCID: PMC5385617.

Human research participants

Policy information about studies involving human research participants

Population characteristics	The inclusion criteria were: 1) 18–64 years old; 2) ability to attend the hospital at regular intervals; 3) ability to independently provide informed consent; 4) BMI between 17.5 and 30.0 kg/m2; 5) daily feeding period of more than 8 hours; and 6) stable body mass (change <±10% of current body mass during the 3 months prior to the study). The exclusion criteria were: 1) night-shift works more than once a week; 2) fasting during the preceding 8 weeks; 3) alcohol consumption more than twice a week; 4) pregnancy, gastrointestinal abnormalities or eating disorders, history of gastrointestinal surgery or systemic disease; 5) use of corticosteroid drugs, β-receptor blockers, or other drugs that might affect the findings; 6) a diagnosis of hypertension, diabetes, or other metabolic diseases; and 7) a diagnosis of insomnia.<br>Among all the participants who finished the trial, 85.7% were female in the eTRF group, 73.1% were female in mTRF group, and 75.0% were female in the control group.<br>All the other characteristics were stated in the manuscript.
Recruitment	Participants were recruited from the Beijing area (China) via posters, emails, flyers, social media, and website advertisements. Ninety participants having a daily feeding period of more than 8 hours and without recent fasting experience were recruited into the trial after signing a written informed consent. Because all the participants were recruited from the Beijing area, they might not have been representative of the wider population. No other potential self-selection bias was present in this trial.
Ethics oversight	Peking Union Medical College Hospital (PUMCH, China, PRC) ethics committee

Note that full information on the approval of the study protocol must also be provided in the manuscript.

Clinical data

Policy information about clinical studies

All manuscripts should comply with the ICMJEguidelines for publication of clinical research and a completedCONSORT checklist must be included with all submissions.

Clinical trial registration	chictr.org.cn : ChiCTR2000029797
Study protocol	Study protocol will be summitted alongside the reporting summary.
Data collection	Participants were screened for eligibility from Feb. 16th, 2020 to Mar. 22nd, 2020, and collected data from Feb. 21st, 2020 to May 26th, 2020.<br>After obtaining signed informed consent from participants, investigators completed forms for each participant to record sociodemographic information, anthropometric measurements, vital signs, percentage body fat measurements, information about medical history, concomitant medication, physical examination, and dietary habits in the research hospital. Metabolic health-related parameters were measured and collected at the beginning and the end of the trial.<br>All the data collection procedures were performed in Peking Union Medical College Hospital. All documents were stored safely in a confidential manner and in accordance with data privacy laws and regulations. Study participants were pseudonymized by using a unique study participant number/code.
Outcomes	Fasting plasma glucose were measured using automated analyzer: AU 5800 by Beckmann-Coulter. Blood Insulin was measured using automated analyzer: ADVIA Centaur XP by Siemens. Insulin resistance index was calculated using HOMA-IR with fasting plasma glucose and fasting blood insulin (HOMA-IR = Glucose x Insulin / 22.5).<br>Body weight were measured using Bioelectrical impedance analyzer HBF-371 (Omron Healthcare Co.) following protocol.

Flow Cytometry

Plots

Confirm that:

- [x] The axis labels state the marker and fluorochrome used (e.g. CD4-FITC).
- [x] The axis scales are clearly visible. Include numbers along axes only for bottom left plot of group (a 'group' is an analysis of identical markers).
- [x] All plots are contour plots with outliers or pseudocolor plots.
- [x] A numerical value for number of cells or percentage (with statistics) is provided.

Methodology

Sample preparation	1. Transfer the EDTA anticoagulated peripheral blood sample into a centrifuge for 10 minutes (2000rpm)<br>2. After centrifugation, the upper plasma was transferred into a 1.5ml EP tube with a 1ml pipette, the remaining was transferred into another EP tube, excess plasma was discarded.<br>3. Add 4ml lymphocyte separation solution into the centrifuge tube, mix the diluted blood cells with a glass pipette, and then slowly add the lymphocyte separation solution along the centrifuge tube wall<br>4. After trimming, centrifuge for 20 minutes. (2500rpm)

5. After centrifugation, use a glass pipette to transfer the middle albuginea layer (Peripheral blood mononuclear cells, PBMC) into a test tube.
6. Add PBS to the test tube and dilute to 10ml, mix well. After trimming, centrifuge for 15 minutes. (2000rpm).
7. After centrifugation, discard the supernatant, add an appropriate amount of PBS solution to dilute and resuspend, then add PBS to 10ml to wash the cells. Centrifuge for 10 min. (1500rpm)
8. After centrifugation, the supernatant was discarded, and the excess PBS solution was aspirated. Resuspend with 500μl PBS.
9. Count the cells and resuspend with 500μl PBS.
10. Add 2×10^6 cells to each tube. Adjust the dyeing volume to about 100μl.
11. Add the corresponding fluorescent antibodies CD4 FITC, CD127 PerCP-Cy5.5, CD25 PE, and CD3 APC to each tube, mix well, and protect from light for 20 minutes.
12. Add 2ml of PBS to each tube, mix well, then centrifuge at 1500 rpm for 5 minutes, and discard the supernatant.
13. Repeat the upper operation, then add 300μl PBS and store at 4°C. Test it on the machine.

Instrument

FACS Canto plus flow cytometer (BD, USA)

Software

FlowJo v10 (BD, USA)

Cell population abundance

Cell sorting not employed

Gating strategy

1 According to the physical characteristics of flow cytometry and the biological characteristics of the cells, FSC-A/FSC-H is used to distinguish adhesive cells, and single cell is located on the diagonal;
2 FSC-A/SSC-A circles the lymphocytes group, Its characteristic is that the FSC-A signal is larger than fragments and smaller than granulocytes, and the SSC-A signal is smaller than granulocytes;
3 According to the biological characteristics of T cells, CD4+ T lymphocytes are circled with CD3 APC/CD4 FITC;
4 According to the biological characteristics and known characteristics of regulatory T cells, in the CD4+ T lymphocyte population, the Treg cell population is further delineated by CD127low/CD25+. The position of negative and positive in the above group is clear, and it is easy to define the boundary of the circle.
A figure exemplifying the gating strategy (Figure S3) will be submitted in a supplemented file alongside the reporting summary.

☒ Tick this box to confirm that a figure exemplifying the gating strategy is provided in the Supplementary Information.
